# Forensic Death Investigations of Dog Bite Injuries in 31 Cats

**DOI:** 10.3390/ani12182404

**Published:** 2022-09-13

**Authors:** Chia-Lin Hsiou, Chih-Chin Hsu, Pei-Wen Liao, Fu-Hua Yang, Ann Nee Lee, Wei-Hsiang Huang

**Affiliations:** Graduate Institute of Molecular and Comparative Pathobiology, School of Veterinary Medicine, National Taiwan University, Taipei City 10067, Taiwan

**Keywords:** canine mitochondrial DNA, dog bite injury, species identification, veterinary forensic science, veterinary forensic pathology

## Abstract

**Simple Summary:**

Fatal dog bite injuries are common in free-ranging cats in Taiwan. The causes of death of these cats are sometimes speculated to be animal cruelty by the general public. To persuade the public and ease speculation, we document and summarize the features of dog bite injuries and further develop a method for canine DNA identification. Performing forensic necropsies on 31 cats with dog bite injuries, we found that puncture wounds, linear or small, round contusions/abrasions, and lacerations/avulsions are characteristic bite-related injuries. A preference for specific body regions was not observed. Using DNA samples from wound swabs and hair remains, we identified canine DNA in 27.3% of cases. This study provides an applicable method for canine DNA identification in cats and reference data for future veterinary forensic investigations of dog bite injuries.

**Abstract:**

Animal bite injuries are common in free-ranging cats in Taiwan, and most fatal animal bite events are presumed to be caused by dogs. However, speculation regarding animal abuse may occur when carcasses with prominent injuries are found by members of the general public. Local animal protection offices and veterinary clinicians sometimes face difficulties in convincing these individuals by identifying specific features of dog bite injuries in cat carcasses. Therefore, the present study analyzed injury patterns and distribution in 31 necropsied cats with animal bite injuries, and applied deoxyribonucleic acid (DNA) analysis for canine DNA identification in 13 cats. The main necropsy findings included puncture wounds (26 (83.9%)), linear or small, round contusions/abrasions (20 (64.5%)), lacerations/avulsions (17 (54.8%)), abdominal wall rupture/laceration (19 (61.3%)), herniation (16 (51.6%)), fractures (21 (67.7%)), broken claws (16 (51.6%)), and hair tufts on the body surface (28 (90.3%)). The most-commonly injured regions were the ventral thorax and axilla (23 (74.2%)), hind limbs (22 (71.0%)), shoulder-to-dorsal thorax (21 (67.7%)), back and flank (20 (64.5%)), abdomen (19 (61.3%)), neck (19 (61.3%)), and hip/tail/perineum (17 (54.8%)). Canine mitochondrial DNA was identified in 3 out of 11 cases (27.3%) that were sampled using wound swabs and in 4 out of 5 cases that had hair entrapped in broken claws. In conclusion, this study determined the distribution and features of dog bite injuries in cats and developed an elemental method using trace evidence for DNA identification in animal bites.

## 1. Introduction

Animal bite injuries can occur in many situations, including animal predation, inter-animal aggression, and organized animal fighting. Among the various animal species of attackers, dogs are the most commonly discussed in veterinary forensic investigations [1]. Dogs may bite other animals for various reasons, such as being threatened during feeding, their territory being invaded, or being in an aggressive or excited mood during estrous [2]. Many animals can be victims of dog bite events, including pets, livestock, poultry, and wild animals, including those kept in captivity. As reported by a large-scale study of trauma in 1000 urban cats and dogs, bite injuries accounted for 10% of traumatic incidents in dogs and 15% in cats, and were the third and second most-common causes of trauma, respectively [3]. In Taiwan, biting injuries (animal interaction) are the third most-common cause of trauma-related deaths in domesticated dogs and cats [4]. Further summarized by recent research, most cats with bite injuries are bitten by dogs [5,6,7]. The commonness of dog bite events therefore makes them a popular issue in both veterinary clinical medicine and veterinary forensic pathology.

In Taiwan, the awareness of animal protection issues is gradually rising. More people in metropolitan areas report dead domestic animals on the streets, especially cats and dogs, to animal protection authorities. When carcasses with prominent external injuries are found, these individuals sometimes claim that the animals died from physical abuse, even without solid evidence. In these situations, local animal protection offices must respond to speculation and determine whether there was human involvement in the death of the animals. However, many of these cats died from animal bite injuries. We presume that the vast majority of fatal animal bite events in cats in urban areas in Taiwan can be contributed to dogs, since most of the native wild carnivores in Taiwan rarely threaten free-ranging domestic cats. Most wild carnivores, such as leopard cats, palm civets, and weasels, have similar or smaller body sizes than cats. Large carnivores, such as Formosan black bears, mainly inhabit mountainous forests, where cats rarely reside [8,9]. Large felids such as clouded leopards are considered locally extinct [10,11], and no native wild canids are found in Taiwan. Furthermore, Taiwanese people in both urban and rural areas tend to feed free-ranging cats and dogs. Therefore, many free-ranging animals have adequate food supplies and fixed feeding spots provided by people. Dog bite events may occur when free-ranging cats and dogs compete for food or when cats try to approach the territory or feeding spots of dogs.

Veterinary clinicians without special training may find it difficult to identify the specific features of dog bite injuries in cat carcasses. Moreover, veterinary clinics and local animal protection offices are not equipped to perform analyses of trace evidence obtained from carcasses, which can be solid evidence of dog bites to provide to people speculating about animal abuse.

The characteristics of dog bite injuries, such as the mechanism of injury formation, common types of injuries, and distribution of injuries, have been reported in several previous studies. Dogs usually attack by biting, shaking, and tearing; thus, a combination of tearing and compressive forces is often the leading cause of tissue damage in dog bite injuries. Tearing force results in laceration or avulsion of the skin and/or deeper tissues. The compressive force of the pointed canine teeth can cause puncture wounds, and the compressive force of the broader and flatter premolar and molar teeth can cause various degrees of crushing injuries, such as contusions, abrasions, and bone fractures [1,12]. Because of dogs’ powerful jaw closure and teeth with rounded apices, dog bite injuries are often consistent with blunt force trauma [13]. More importantly, puncture wounds are not necessarily present in every dog bite injury due to the rounded apices of their teeth, which may not penetrate the skin in some cases [1]. Therefore, dog bite injury is famous for its “iceberg” feature, which means that it has relatively minor skin lesions that conceal the severe laceration and damage of deeper tissues and organs [5,7,12,14,15]. This feature may be due to the loose subcutaneous tissue and mobile skin of cats and dogs, which allow the teeth to effortlessly move and tear the underlying tissue [7,15,16,17,18,19].

Regarding the distribution of dog bite wounds and the preferred targets of body regions, different opinions have been reported in previous research. One study indicated that dogs bite any area they can [1], while several other studies concluded that there was a common distribution of dog bite injuries in specific regions of the body [7,13,18]. Few studies have documented the distribution of dog bite injuries in cats, and cats experience fewer dog bite events [7,18,20]. Although conclusive agreement regarding the common distribution of dog bite injuries has not been reached, many of these previous studies mentioned that the distribution of bite injuries might be affected by the size of victims, and cats and small dogs were more likely to have multiple injuries on their bodies [5,7,18,20]. We presume that the reason for attacking and the relative size difference between a dog and its victim affect the patterns and distribution of dog bite injuries, thus making this topic diverse and complicated. In addition, most previous research investigating clinical dog bite cases focused more on wound management and outcomes. Little is known about the distribution of dog bite injuries in necropsy cases in veterinary forensics.

Several methods can be used to identify species of attackers in animal bite events. Witness accounts are useful but are not always available. Two important pieces of trace evidence on the bodies of victims can be used for deoxyribonucleic acid (DNA) analysis: the saliva and hair remains of the attacker. First, the saliva of the attacker might be deposited on the coat, body surface, and wounds of the victim [21]. It has also been reported that the hair of the victim is sometimes stuck together by the attacker’s saliva, forming clumps or tufts of hair, which can provide DNA trace evidence for identifying the attacker [21,22,23]. Second, broken or frayed claws in animal bite events may occur when there is a violent interaction between the victim and the attacker, such as when the victim struggles to escape or defend itself, the victim is chased by the attacker, or the attacker drags the victim [21]. Hairs of the attacker might become entrapped in the victim’s broken claws when it struggles during the attack [21]. This finding was observed in a study of coyote attacks in cats; however, DNA analysis of the hair remains in claws has not yet been performed [22].

A few previous studies have successfully sampled attacker saliva DNA from bite wounds. In a study on coyote predation on sheep, samples were taken only from puncture wounds with skin reflection to prevent collecting saliva from scavengers who consume the victims after their death [24]. Other studies emphasized the importance of avoiding contamination of the blood of victims when sampling [21,23,25,26], since bleeding may wash off the saliva DNA [21]. However, methods performed in previous research might not be adaptable in different situations [26]. To conduct DNA analysis of the saliva on victims, mitochondrial DNA (mtDNA) is suggested, since the saliva DNA might be damaged, degraded, and be of low quality, making nuclear DNA difficult to detect. On the other hand, mtDNA has a higher copy number per cell and is more resistant to degradation than nuclear DNA [21,27]. Moreover, a previous study on mutilation of cats by foxes successfully sampled fox mtDNA on cat fur [23].

Hairs of attackers entrapped in broken claws are another type of trace evidence that can be used to identify the species of attackers in cases of animal bite injuries. Hair-forming cells in the hair bulb contain abundant mitochondria that are also deposited in the hair shaft. Therefore, mtDNA analysis is useful in hair samples [28]. In some wild-animal mortality investigations, hairs have been used as one of the clues for predator identification [29,30]. However, to the best of our knowledge, no studies to date have specifically investigated the DNA of hair remains entrapped in cat claws in animal attack events.

Trying to delve into the issue of dog bite injuries in cats with an analytical and academic outlook, local animal protection offices started collaborating with the Laboratory of Veterinary Forensic Medicine and Comparative Pathology at the Graduate Institute of Molecular and Comparative Pathobiology, National Taiwan University, in 2019. To relate to the local situation in Taiwan, the present research aimed to analyze the patterns of dog bite injuries in cats by performing necropsy and applying trace evidence analysis for canine DNA identification.

## 2. Materials and Methods

### 2.1. Case Collection

Forensic necropsy was performed on all cats submitted to the Laboratory of Veterinary Forensic Medicine and Comparative Pathology at the Graduate Institute of Molecular and Comparative Pathobiology, School of Veterinary Medicine, National Taiwan University, between 2019 and 2020. All cases were either carcasses found on the street or animals found injured on the street that were rescued by animal protection offices but eventually died or were euthanized in animal hospitals. After the necropsies, cases with bite-related wounds were included in this study. For all cases included in this study, the sex, breed, and age were recorded and reported, and representative specimens were collected for further DNA analysis (see below).

### 2.2. Necropsy and Injury Patterns Analysis

Histories of the carcasses, including medical histories, witness accounts of the events, and claims of the reporting individuals, were recorded on submission forms sent from animal protection offices. We categorized their histories according to these submission forms. Before the carcasses were approached, we recorded the species, sex, estimated age, fur color, microchip number, and post-mortem conditions. The body conditions of the carcasses were determined by the veterinarians who performed necropsy. Body condition was summarized according to the nine-point body condition scoring (BCS) system: BCS 1–2 = emaciated, BCS 3 = underweight, BCS 4–5 = normal, BCS 6–7 = overweight, and BCS 8–9 = obese.

During the necropsies, the head, oral cavity, perineum, and four body views (dorsal–ventral, ventral–dorsal, right lateral, and left lateral) were routinely examined and photographed. The body surface and claws were then examined in detail. In body regions suspected of traumatic injuries, hair was shaved using a scalpel to expose the skin surface and edges of the wounds. After shaving, close-up views of each finding and the four body views were photographed.

The bodies were opened using a midline incision, and organs were removed from the tongue to the rectum as one block (“en masse” removal). The organ blocks were separated into individual organs. Body fluids and gastric contents were routinely measured.

All necropsy findings were recorded and marked on anatomical diagrams in the forensic necropsy records. In particular, we focused on the types, shapes, depths, and characteristics of traumatic injuries. Fatal external injuries and suspected hemorrhagic lesions were sampled. All organs, including the brain, were routinely examined and sampled for histopathological analysis. In forensic necropsy reports, the external, internal, and histopathological findings were described in detail, and the cause of death was stated in each case. 

Injury pattern analysis was conducted by performing necropsies and reviewing the necropsy reports, records, and gross photographs. Features and necropsy findings were listed and categorized for analysis. Ten anatomical body regions were defined to analyze the distribution of injuries, including the head, neck, shoulder-to-dorsal thorax, forelimb, ventral thorax and axilla, abdomen, back and flank, inguinal regions, hip/tail/perineum, and hindlimbs.

### 2.3. DNA Sample Collection

To detect DNA from attackers during animal bite events, we collected two types of DNA samples: hair remains and wound swabs.

First, the claws of all extremities were routinely examined during the external examination. When hair remains were observed in broken claws, photographs were taken, and the locations were documented in necropsy records.

Second, since 2020, we have collected cotton swab samples from bite wounds that were highly suspected of animal bite injury during necropsies. Two areas of samples were collected: (1) swabs from around the wound and (2) swabs from the deeper part of the puncture or laceration wound. If a puncture or laceration wound was found after hair shaving, swabs around the wound were not taken.

All DNA samples were dried for at least one hour, placed into paper envelopes, and stored at −20 °C until analysis.

### 2.4. DNA Extraction and Polymerase Chain Reaction

DNA was extracted from cotton swabs using PrepFiler^®^ and PrepFiler^®^ BTA Forensic DNA Extraction Kits (Thermo Fisher Scientific, Waltham, MA, USA) following the manufacturer’s protocols. The final elution volume was 50 µL. For hair remains, three to five 0.5–1 cm pieces of hair with roots were cut for DNA extraction. A DNeasy^®^ Blood & Tissue Kit (QIAGEN, Hilden, Germany) was used to extract DNA following a user-developed protocol provided by the manufacturer, in which 20 μL of 1M dithiothreitol (Thermo Fisher Scientific, Waltham, MA, USA) was added to the lysis buffer. The DNA samples (100 µL) were eluted in the final step. All extracted DNA samples were stored at −20 °C before polymerase chain reaction (PCR).

Canine-specific primers (forward 5′ CCTTACTAGGAGTATGCTTG 3′ and reverse 5′ TGGGTGACTGATGAAAAAG 3′) targeting a 100 bp region in the canine mitochondrial cytochrome b gene, designed by Rahman et al. [31], were used in this study. PCR was performed in a thermocycler (Labcycler Basic & Labcycler Gradient, SensoQuest GmbH, Göttingen, Germany) using 20 µL of reaction mixture consisting of 10 µL of amaR OnePCR™ reagent (GeneDireX, Las Vegas, NV, USA), 1 µL of each primer (forward and reverse), 2 µL of DNA template, and 6 µL of double-distilled water. The PCR cycling conditions were as follows: initial denaturation at 94 °C for 3 min with 40 cycles of denaturation (94 °C for 30 s), annealing (54 °C for 30 s), and extension (72 °C for 30 s). The final extension step was performed at 72 °C for 5 min. Gel electrophoresis was performed to detect PCR products. The positive bands observed under ultraviolet light were cut. The cut blocks of the agarose gel were sent to Tri-I Biotech Inc. (Taipei, Taiwan) for DNA sequencing. DNA sequencing results were then searched using the Basic Local Alignment Search Tool (BLAST) in the National Center for Biotechnology Information (NCBI) database.

## 3. Results

### 3.1. Characteristics of Cases

Between 2019 and 2020, 166 cats underwent forensic necropsy. Among these cats, 89 (53.6%) experienced blunt force trauma. Of the cats with blunt force trauma, 31 (34.8%) with animal bite injuries were included in this study. The majority of these cases (26 (83.9%)) were provided by the Taipei City Animal Protection Office, followed by the animal protection offices of Taichung City (3 (9.7%)), Taoyuan City (1 (3.2%)), and Pingtung County (1 (3.2%)). Only five dogs had animal bite injuries between 2019 and 2020 and were not included in this study.

Witness accounts of dog bite events were unavailable in most cases (26/31 (83.9%)). The majority of cases (30/31 (96.8%)) were found dead on the street or died soon after rescue and hospitalization. Only 1 cat (3.2%) was euthanized after hospitalization. All 31 cats (100%) were identified as mixed-breed cats. Among the cats, 12 (38.7%) were spayed females, 7 (22.6%) were intact males, 7 (22.6%) were intact females, and 5 (16.1%) were castrated males. Twenty-two cats (71.0%) were identified as adults, 6 (19.3%) as kittens, and 3 (9.7%) as juveniles. The mean weight was 3.2 ± 1.7 kg, and 18 cats (58.1%) had normal body condition, 7 (22.6%) were overweight, 3 (9.7%) were obese, 2 (6.4%) were underweight, and 1 (3.2%) was emaciated.

### 3.2. Distribution and Types of Injuries

**Figure 1:**
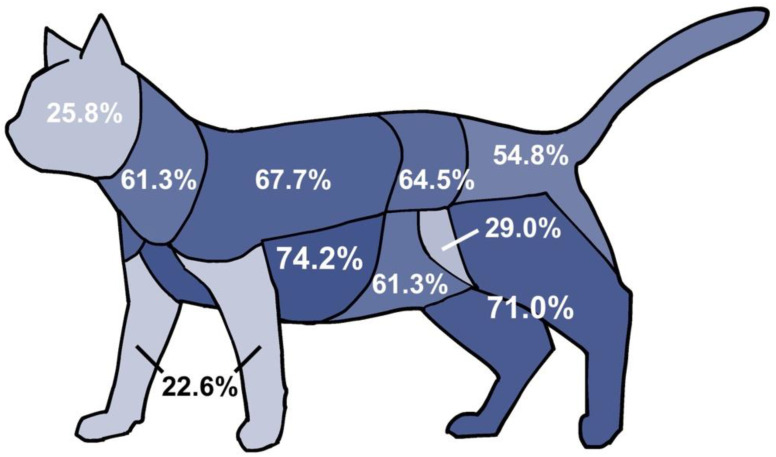
Illustration of the distribution of soft tissue injuries in different body regions of cats.

Injuries to the skin, subcutis, and/or muscles were classified as soft tissue injuries and categorized into 10 body regions in this study. The distribution of soft tissue injuries is shown in Figure 1. Multiple injuries involving more than one body region were observed in all cases (31 (100.0%)). The most-commonly injured regions were the ventral thorax and axilla (23 (74.2%)), hindlimbs (22 (71.0%)), shoulder-to-dorsal thorax (21 (67.7%)), back and flank (20 (64.5%)), abdomen (19 (61.3%)), neck (19 (61.3%)), and hip/tail/perineum (17 (54.8%)). Only one cat (3.2%) had missing body parts.

**Figure 2:**
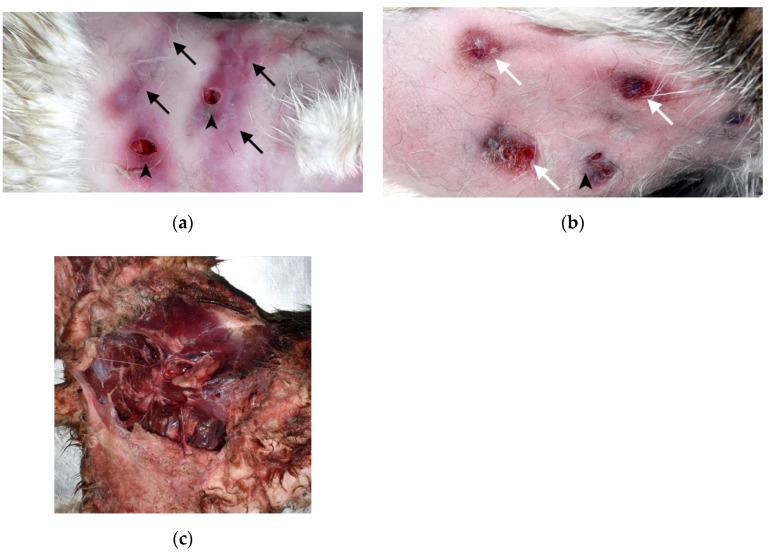
Bite-related injuries in cat carcasses: (**a**) Puncture wounds (arrowheads) and linear abrasions with contusions (black arrows) on the skin of the shoulder-to-dorsal thorax region. The linear abrasions with contusions may be tooth marks caused by incisors when the skin was dragged. (**b**) Puncture wounds (arrowhead) and small, round contusions with abrasions (white arrows) on the skin of the lumbar region. (**c**) Massive laceration and avulsion of the skin and skeletal muscles of the inguinal to hindlimb regions.

External injuries were further classified into specific types. Puncture wounds, linear or small, round contusions/abrasions, and lacerations/avulsions were considered bite-related injuries (Figure 2a–c). Puncture wounds were present in 26 cats (83.9%), linear or small, round contusion/abrasion wounds were present in 20 cats (64.5%), and laceration/avulsion wounds were present in 17 cats (54.8%). Only five cats (16.1%) had paired puncture wounds, which can be created by the canine teeth of attackers. All 31 cats had injuries that could be classified as at least one of these bite-related injuries.

**Figure 3:**
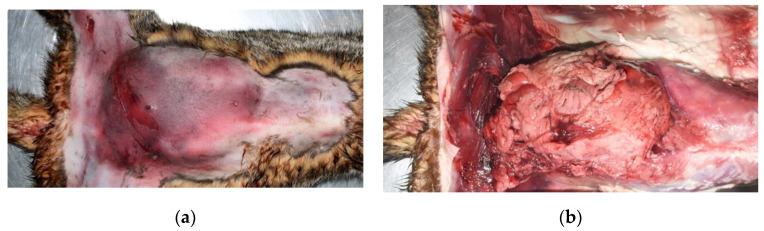
The “iceberg” feature of a dog bite injury to the abdomen of a cat. (**a**) After hair shaving, only swelling and massive contusion on the skin of the abdomen is noted. (**b**) After skin reflection, the abdominal cavity and subcutis herniation of the intestine and omentum from the lacerated abdominal wall can be identified.

The “iceberg” feature was seen in 23 cats (74.2%). These cats only had puncture wounds, small lacerations, and/or nonpenetrating wounds, such as abrasions and contusions, on the skin; however, after skin reflection, massive subcutaneous hemorrhage, lacerations of skeletal muscles and/or body walls, and herniations of organs could be observed (Figure 3a,b). The eight cats that did not exhibit the “iceberg” feature had exposed organs, massive skin laceration, and/or missing limbs.

The common types of internal injuries were abdominal wall rupture/laceration (19 (61.3%)), hernia (any site) (16 (51.6%)), thoracic wall rupture/laceration (15 (48.4%)), and abdominal organ rupture (any organ) (15 (51.6%)) (Table 1). The liver was the most-commonly ruptured abdominal organ (11 (35.5%)), and abdominal–subcutaneous hernia was the most common type of hernia (12 (38.7%)). Diaphragm rupture was seen in six (19.4%) cases, and all of the rupture sites were at the muscular portions of diaphragms.

Fractures were present in 21 cats (67.7%) (Table 2). The most common types were rib fractures (any site) (15 [71.4%]), followed by vertebral arche/process fractures (4 [19.0%]), vertebral body fractures (3 [14.3%]), and pelvic fractures (3 [14.3%]). The lumbar vertebrae were the most affected sites for all vertebral fractures. Rib fractures were more commonly seen in the bodies of ribs than other parts.

### 3.3. Other Necropsy Findings

**Figure 4:**
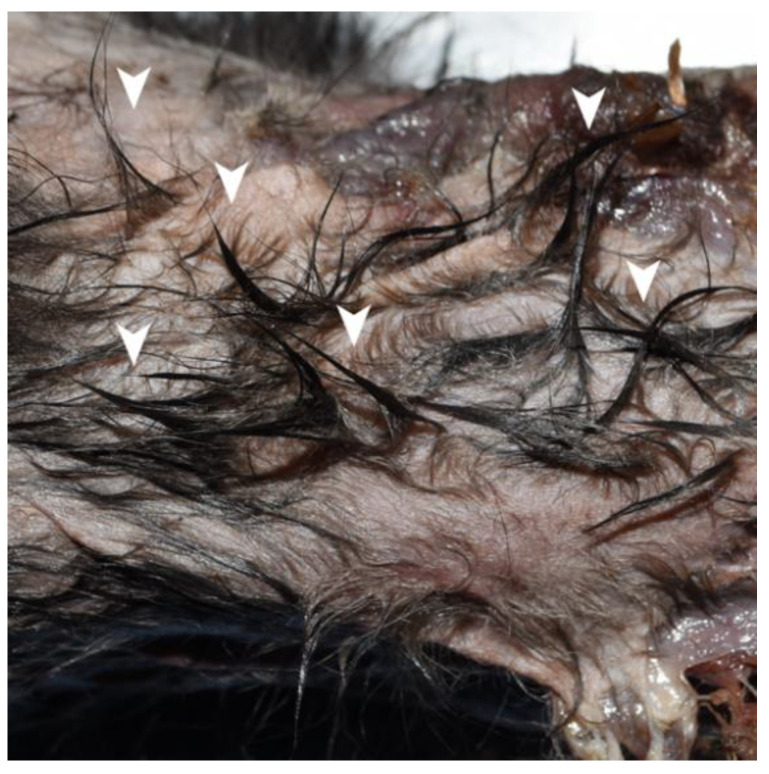
Hair tufts in a cat carcass. Hair tufts (arrowheads) are presumably stuck together by the saliva of the attacker.

**Figure 5:**
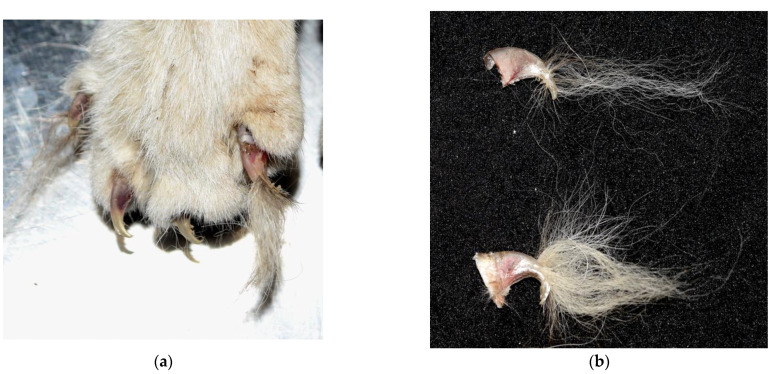
Broken claws with entrapped hair remains in the right forelimb of a cat. (**a**) Long, white hair remains entrapped in the broken claws of a cat. The cat was short-haired and had a tabby and white coat color. (**b**) Sampled broken claws with entrapped hair remains. The hair remains tested positive for canine mitochondrial DNA (Case 4).

Twenty-eight cats (90.3%) had hair tufts (Figure 4) present in areas without observable blood. These hair tufts were presumed to be stuck together with saliva. Broken claws were observed in 16 (51.6%) cats. Of these cats, five (31.3%) had suspected hair remains entrapped in their broken claws (Figure 5a,b).

**Figure 6:**
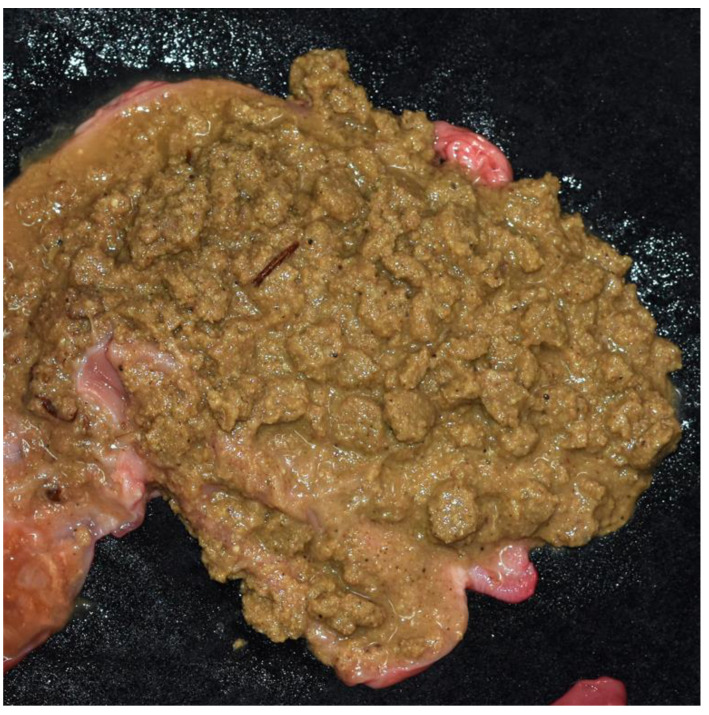
Gastric contents in the stomach of a cat. A large amount of gastric content with undigested food remains, which were identified as kibbles. The kibbles were considered human-provided food.

More than half of the cats (16 (51.6%)) had a large amount of gastric content, usually consisting of undigested food (Figure 6). In this study, human-provided kibbles were detected. 

Finally, only one cat had missing limbs. The mesentery was intact in all cats with exposed intestines.

### 3.4. Canine Mitochondrial DNA Detection

The sample sites and results of wound swabs are shown in Table 3. A total of 42 wound swabs were collected from 11 cases. All swabs were taken from bite-related wounds. Nine out of 42 swabs (21.4%) and 3/11 cases (27.3%) tested positive for canine mtDNA.

The results for hair remains in claws are listed in Table 4. In total, 5 cases and 10 samples were tested. Five out of 10 samples (50%) and 4/5 cases were positive for canine mtDNA and were successfully sequenced.

In brief, canine mtDNA was identified in 6/13 sampled cases (46.2%) and 6/31 cats (19.4%). In combination with the necropsy findings, the attackers in these cases were confirmed to be dogs.

## 4. Discussion

In this study, we analyzed the patterns and distributions of animal bite injuries in necropsied cats and applied canine DNA identification to confirm dog bites. Characteristics of dog bite injuries, including bite-related injuries and the “iceberg” feature, were identified. Moreover, we demonstrated a primary method for canine mtDNA identification in dog bite injuries in cats by analyzing wound swabs and hair remains. Based on the features of necropsy findings and the results of DNA identification, we concluded that dogs were the main attackers in animal bite events in cats in Taiwanese urban areas. This study highlights the importance of injury patterns and trace evidence analyses in veterinary forensic investigations.

Only cats were included in this study because we received a greater number of cats (31) than dogs (5) with animal bite injuries; however, in previous research, dog bite events were reported less frequently in cats [7,18,20]. A probable reason for this is that cats have a higher risk of dying from animal bite events, while previous research usually focused on live animals who suffered dog bites. The severity and number of injured body areas affect the survival rate of cats with dog bite wounds. In addition, cats were more likely to have multiple injuries, and 93% of fatal cases had injuries to the abdomen, thorax, or both [5]. In our study, injuries to both the thorax and abdomen were frequently present (ventral thorax and axilla, 74.2%; abdomen, 61.3%), and all of our cases sustained multiple injuries. Moreover, all our cases were cats that died or became immobile on the street because of severe injuries caused by animal bites. Cats and dogs that survived dog bites, who were presumed to have milder injuries, were not included in this study. Thus, the prevalence of cats in our study might be due to the higher risk of severe injuries and death in cats.

Regarding whether dogs have preferred targets of body regions or they bite any area they can, different opinions have been expressed in previous research [1,7,13,18]. The most-commonly injured regions of cats with dog bite injuries in previous studies were the back and extremities [18] or the back, thorax, and abdomen [7]. For smaller victims, such as cats, dogs might prefer targeting the chest and neck [13]. Overlapping and similarities were seen between our results and those of previous studies. The ventral thorax and hindlimbs were the most-commonly injured regions in our results, and injuries to many other body regions (such as shoulder-to-dorsal thorax, back and flank, abdomen, neck, and hip/tail/perineum) were also present in over 50% of our cases. A preference for specific regions was not observed. This indicates that our results support the theory that dogs tend to bite any area they can.

We categorized puncture wounds, linear or small, round contusions/abrasions, and lacerations/avulsions as bite-related injuries. The patterns and characteristics of these dog bite injuries were compatible with those described in previous studies [1,12]. Puncture wounds were observed in 83.9% of our cases. This finding indicates that puncture wounds are not necessarily present in every dog bite injury due to the rounded apices of teeth, which may not always penetrate the skin [1]. When teeth do not penetrate the skin, patterns such as linear or small, round contusions/abrasions can be left by teeth when biting, scratching, and sliding on the skin. Moreover, the “iceberg” feature mentioned in previous studies [1,5,7,12,15] was observed in 74.2% of our cases. This reflects not only the characteristic rounded apices of teeth, but also the classical behavior of shaking and tearing when dogs bite, which results in relatively minor lesions on the skin and severe lacerations/avulsions in deeper tissue. The high percentages of herniation (51.6%) and thoracic (48.4%)/abdominal (61.3%) wall ruptures in our results can also be related to shaking and tearing. We recommend that hair shaving and skin reflection is performed when conducting necropsies on suspected dog bite victims to expose skin lesions and examine deeper tissues.

Fractures were present in 67.7% of cats in this study, and the ribs were the most common fracture site. Considering the powerful jaw closure of dogs that causes crushing injuries [1,12], it is reasonable that rib fractures can occur when dogs bite cats’ chests. In addition, the ventral thorax and shoulder-to-dorsal thorax were both highly affected body regions in our results of the distribution of soft tissue injuries, which might explain the commonness of rib fractures.

In this study, only one cat had missing limbs, and the mesentery was intact in cats with exposed intestines. These findings further support our theory that most of the attackers were dogs, because their behaviors are different from those of predators that hunt for food or scavenge prey. Predators, such as coyotes and foxes mentioned by previous studies, commonly mutilate cats and leave incomplete carcasses, and the injury patterns may vary among predators because of variations in feeding habits [22,23]. In contrast, stray dogs (lost or abandoned pet dogs) and roaming dogs (pet dogs who are free to roam) do not hunt for food. They depend mainly on human food sources or garbage. Thus, as shown in our results, they rarely consume prey, and leave mutilated carcasses. Furthermore, since these dogs have poor hunting skills, the wounds that they cause in other animals are more extensive [32], which may explain the high prevalence of multiple injuries in our results. It has also been suggested that stray and roaming dogs, among all types of dogs, contribute to most of the killings and attacks of other animals [32].

A new characteristic of dog bite injuries was identified in our study. Of the 31 necropsied cats, 51.6% had a large amount of gastric content, indicating that they had eaten a meal shortly before death. This finding may verify our opinion regarding the reasons for dog attacks in Taiwan. Many free-ranging dogs in Taiwan have food supplies and fixed feeding spots provided by people claiming to be the caretakers of street animals. We suspect that dog bite events may occur when new members or other street animals try to approach the territory or feeding spots of these free-ranging dogs. In addition, there is some overlap in the feeding and activity spots of street cats and dogs, since the caretakers of street animals also feed street cats. When cats eat the provided food in fixed feeding spots, they become the targets of dogs. We also identified human-provided kibbles in the stomachs of cats (Figure 6).

In this study, 51.6% of the cats had broken claws, and 13.9% had hair remains entrapped in their broken claws. Canine mtDNA was successfully identified in the hair remains of four cats. Thus, we highly recommend that routine and detailed examination of claws and sampling of probable hair remains is performed when examining carcasses with suspected animal bite injuries. In addition, canine mtDNA was detected in 9/42 wound swabs (3/11 cases). One case showed positive results for both wound swabs and hair remains. The combination of necropsy findings and positive results for canine DNA provides solid evidence of dog bites in these cats.

We observed hair tufts stuck together with the saliva of the attacker in 90.3% of the cats. A considerable amount of canine saliva remained on the cats. However, the positivity rate of swab samples was lower than that of hair remains. This low positive rate may be related to sample collection, the conditions of the samples, and/or the primers. First, only 11 cases had been sampled since the routine collection of wound swabs began in 2020. Second, most carcasses in this study were preserved by freezing, and were defrosted before necropsy. Once defrosted, humidity can be high while preserving the bags. Furthermore, many carcasses were collected after several hours of street exposure. It is conceivable that the salivary DNA in the wounds was severely damaged and degraded in our cases. Third, the canine-specific primers used in this study were originally designed for detecting breakdown mtDNA in processed meat [31]. We chose these primers due to their short target regions and the similar harsh condition of our samples compared to that of processed meat. Although these primers were proven to be canine specific in our experiment, they were not explicitly used to detect mtDNA in hair and saliva. An improved DNA extraction method and the development of sensitive and specific primers for detecting DNA in hair and wound swabs are required. Additionally, an advanced DNA typing method for animal bite injuries to identify individual attackers should be developed in the future.

One cat in this study was primarily classified as having an undetermined cause of trauma due to unusually severe injuries; however, canine DNA was detected in the wound swabs after performing DNA analysis (Case 1 in Table 3). The cat had comminuted facial fractures, abrasions, multifocal lacerations, puncture wounds, and missing limbs with comminuted fractures at the mutilated sites. The hairs around the mutilated sites were stuck together and formed tufts. The cat was initially reported as an animal abuse case by a person who first discovered the carcass. This person described the wounds as sharply cut, human-made wounds. The speculation of animal cruelty escalated further on social media and attracted attention. Similar phenomena on social media were also mentioned in a study on cats dismembered by coyotes and a study on cats dismembered by foxes [22,23]. This particular case points out the importance of performing forensic necropsy and DNA analysis in this kind of dismembered carcass, since findings suggestive of animal bite injury might be identified afterward. If none of the findings support animal bite injury, non-accidental injury and other accidental causes of trauma should be considered.

This study has some limitations. First, the findings were restricted to fatal cases and necropsied cats. Dog bite injuries in clinical cases and surviving cases may have different features from our results. Second, the causes of dog bite injuries we discussed might not be representative of the whole situation in Taiwan, since the majority of cases in this study were submitted by animal protection offices in urban and suburban areas.

In this study, we have referred to cats as the “victims” of dog bite injuries, but it should be noted that cats can also be attackers in animal bite events. Native animals, such as small mammals and birds, may fall victim to free-ranging cats [33,34]. The injury patterns of cat bite wounds in these wild animals and the identification of feline DNA are worth studying. Other larger native animals in Taiwan, such as pangolins, Reeve’s muntjacs, and leopard cats, can also be the victims of dog bite injuries [34]. Leopard cats are an endangered species in Taiwan. Due to the development of roads in the natural habitat of leopard cats, they are now threatened by cars and dog bite events following the invasion of domestic dogs into their habitat [35,36]. Some people even train their dogs to hunt leopard cats [36]. Since leopard cats share similar body sizes with domestic cats, our results might be helpful for studying dog bite injuries in leopard cats in the future.

## 5. Conclusions

The present study investigated the distribution and patterns of dog bite injuries in cats, demonstrated findings related to local circumstances in Taiwan, and developed a primary but applicable method for identifying canine DNA in cats. The results could serve as reference data for future veterinary forensic investigations of mysterious animal deaths in urban areas where animals interact with stray or roaming dogs. The study underscored the importance of veterinary forensic necropsy and shed light on veterinary forensic research regarding injury pattern analysis. 

## Figures and Tables

**Table 1:** Number and percentage of internal injuries in 31 cats.

Internal Injuries		Number of Cats (%)
Head		
Massive brain injury		0
Partial brain injury		4 (12.9)
Eyeball luxation/rupture		0
Neck		
Trachea/larynx cartilage rupture		1 (3.2)
Thorax		
Thoracic effusion		12 (38.7)
Thoracic effusion with hematoma		3 (9.7)
Thoracic wall rupture/laceration		15 (48.4)
Heart rupture		1 (3.2)
Heart contusion		1 (3.2)
Pericardial rupture		0
Hemopericardium		1 (3.2)
Lung rupture		10 (32.3)
Lung contusion		1 (3.2)
Abdomen		
Abdominal effusion		4 (12.9)
Abdominal effusion with hematoma		4 (12.9)
Diaphragm rupture		6 (19.4)
Abdominal wall rupture/laceration		19 (61.3)
Abdominal organ rupture	Any	16 (51.6)
	Liver	11 (35.5)
	Spleen	6 (19.4)
	Kidney	4 (12.9)
	Adrenal gland	1 (3.2)
	Urinary bladder	0
	Stomach	3 (9.7)
	Small intestine	1 (3.2)
Abdominal organ contusion	Any	6 (19.4)
	Retroperitoneal fat	2 (6.5)
	Kidney	4 (12.9)
	Omentum/mesentery	1 (3.2)
	Adrenal gland	2 (6.5)
	Urinary bladder	0
	Liver	0
	Spleen	1 (3.2)
	Stomach	1 (3.2)
	Diaphragm	0
Large blood vessel rupture	Any	5 (16.1)
	Abdominal aorta	2 (6.5)
	Thoracic aorta	1 (3.2)
	Pulmonary artery	0
	Thoracic CVC	1 (3.2)
	External jugular vein	0
	Abdominal CVC	1 (3.2)
Hernia	Any	16 (51.6)
	Thorax–abdomen	2 (6.5)
	Abdomen–subcutis	12 (38.7)
	Abdominal wall	3 (9.7)
	Other	0

Abbreviation: CVC, caudal vena cava.

**Table 2:** Number and percentage of fractures in 21 cats.

Types of Fracture		Number of Cats (%)
Scapula		2 (9.5)
Rib	Any	15 (71.4)
	Single	6 (28.6)
	Unilateral multifocal	5 (23.8)
	Bilateral multifocal	4 (19.0)
Sternum		1 (4.8)
Vertebral body	Any	3 (14.3)
	Cervical	0
	Thoracic	0
	Lumbar	3 (14.3)
	Sacral/coccygeal	0
Vertebral arches/processes	Any	4 (19.0)
	Cervical	1 (4.8)
	Thoracic	0
	Lumbar	3 (14.3)
	Sacral/coccygeal	0
Pelvis		3 (14.3)
Extremities	Any	1 (4.8)
	Forelimbs	1 (4.8)
	Hindlimbs	1 (4.8)
Head		1 (3.2)

**Table 3:** PCR and BLAST results of wound swabs.

Case Number	Sample Site	PCR Result	Result Species	Highest Percent Identity
Case 1	1. Rt. FL mutilated site (deep)	Positive (weak band)	No significant similarity found	
	2. Rt. FL mutilated site (surface)	Positive (weak band)	No significant similarity found	
	3. Rt. HL mutilated site (surface)	Positive (weak band)	*Canis lupus familiaris*	98.61%
	4. Lt. FL mutilated site (deep)	Negative		
	5. Lt. FL mutilated site (surface)	Negative		
	6. Head laceration (deep)	Positive (weak band)	*Canis lupus familiaris*	97.22%
Case 2	1. Lt. mandible puncture wound (surface)	Negative		
	2. Lt. mandible puncture wound (deep)	Negative		
Case 3	1. Rt. hip puncture wound	Negative		
	2. Lt. flank puncture wound (surface)	Negative		
	3. Lt. flank puncture wound(deep)	Negative		
Case 4	1. Lt. puncture wound (deep)	Negative		
	2. Rt. HL puncture wound (deep)	Negative		
	3. Lt. hip puncture wound	Negative		
Case 5	1. Lt. pelvis puncture wound	Negative		
	2. Lt. HL puncture wound	Negative		
	3. Rt. chest and axillary puncture wound	Negative		
Case 6	1. L3 puncture wound (deep)	Negative		
	2. Lt. orbit puncture wound	Negative		
	3. L4 puncture wound	Negative		
	4. Lt. HL puncture wound	Negative		
	5. Lt. HL puncture wound (deep)	Negative		
Case 7	1. Lt. flank puncture wound (deep)	Negative		
	2. Lt. flank puncture wound (deep)	Negative		
Case 8	1. Rt. trunk puncture wound	Positive (weak band)	*Canis lupus familiaris*	97.22%
	2. Rt. abdomen puncture wound	Negative		
	3. Lt. pelvis puncture wound	Positive (weak band)	*Canis lupus familiaris*	97.26%
	4. Rt. trunk smaller puncture wound	Positive (weak band)	*Canis lupus familiaris*	97.22%
	5. Rt. HL puncture wound	Negative		
	6. Dorsal pelvis puncture wound	Positive (weak band)	*Canis lupus*	97.18%
Case 9	1. Ventral neck puncture wound	Negative		
	2. Lt. clavicle puncture wound	Negative		
	3. Rt. humerus puncture wound	Negative		
	4. Rt. scapula puncture wound	Negative		
	5. Rt. abdomen puncture wound	Positive (weak band)	*Canis lupus familiaris*	94.52%
	6. Rt. inguinal region puncture wound	Negative		
	7. Rt. dorsal pelvis puncture wound	Negative		
	8. Dorsal sacrum puncture wound	Negative		
Case 10	1. Dorsal hip puncture wound	Negative		
	2. Left tail base puncture wound	Negative		
Case 11	1. Rt. HL lateral hock puncture wound	Negative		
	2. Rt. HL medial hock puncture wound	Negative		

Abbreviation: Rt., right; Lt., left; FL, forelimb; HL, hindlimb; L3, the third lumbar vertebra; L4, the fourth lumbar vertebra.

**Table 4:** PCR and BLAST results of hair remains in claws.

Case Number	Sample Site	PCR Result	Result Species	Highest Percent Identity
Case 3	Lt. HL 3rd digit	Negative		
Case 4	Rt. FL 2nd digit-1	Negative		
	Rt. FL 2nd digit-2	Negative		
	Lt. FL 3rd digit	Negative		
	Lt. FL 4th digit	Negative		
	Rt. FL 5th digit	Positive (weak band)	*Canis lupus familiaris*	100%
Case 9	Lt. HL 1st digit	Positive	*Canis lupus familiaris*	97.06%
	Lt. FL 5th digit	Positive	No significant similarity found	
Case 12	Rt. FL 2nd and 3rd digits	Positive (weak band)	*Canis lupus familiaris*	97.14%
Case 13	Bilateral FL 2nd and 4th digits	Positive (weak band)	*Canis lupus familiaris*	97.26%

Abbreviation: Rt., right; Lt., left; FL, forelimb; HL, hindlimb.

## Data Availability

Not applicable.
